# Smartphone as a tool for evaluating oblique muscle dysfunction

**DOI:** 10.5935/0004-2749.20220041

**Published:** 2022

**Authors:** Yessa Vervloet Bertollo Lamego Rautha, Luis Eduardo Morato Rebouças de Carvalho, Marcelo Francisco Gaal Vadas, Carlos Fumiaki Uesugui, Laís Yumi Sakano, Ronaldo Boaventura Barcellos

**Affiliations:** 1 Setor de Estrabismo, Departamento de Oftalmologia, Universidade de São Paulo, São Paulo, SP, Brazil; 2 Setor de Estrabismo, Departamento de Oftalmologia, Irmandade da Santa Casa de Misericórdia de São Paulo, São Paulo, SP, Brazil

**Keywords:** Strabismus, Oculomotor muscle, Anisotropy, Smartphone, Cell phone, Estrabismo, Músculo oculomotor, Anisotropia, Smartphone, Telefone celular

## Abstract

**Purpose:**

The aim of this study was to describe a simple, accessible, and reliable method using a smartphone for evaluating oblique muscle dysfunctions.

**Methods:**

The photograph rotation tool in the iPhone PHOTO app was used by 75 examiners to evaluate 22 photographs from only 9 patients, captured in infra-and supra-dextroversion, and infraand supra-levoversion, as not all the patients were photographed in the 4 positions mentioned. Each patient received a score for the superior and inferior oblique muscle functions, ranging from -4 (hypofunction) to 4 (hyperfunction) or 0 (normal function), using preediting and postediting photographs. These values were compared with the scores previously given by trained personnel in strabismus screening. The difference in score between the two groups was expressed in natural (whole and non-negative) numbers. The mean and pattern deviation were then calculated.

**Results:**

The scores of most of the edited photos showed a lower mean than those of the unedited ones, except for a patient with left superior oblique hyperfunction. The patients with no oblique dysfunction and those with right superior oblique hyperfunction demonstrated (after editing the photograph) scores with greater similarity with their initial scores (p<0.05 and p<0.01, respectively). Similar results were found in the patients with oblique hypofunctions and right inferior oblique hyperfunction (p<0.01).

**Conclusion:**

The proposed method for assessing muscular function in vertical strabismus is reproducible, accessible, simple, and reliable, and provides better consistency to the admeasurement.

## Abstract (pt)

**Objetivo:**

Descrição de um método simples, acessível e confiável para a medida das disfunções dos músculos oblíquos, utilizando-se *smartphone*.

**Métodos:**

Foi utilizado o recurso de rotação de fotografias do aplicativo FOTOS do iPhone^®^; 75 examinadores avaliaram 22 fotos de 9 pacientes, obtidas em infra e supra dextroversão, infra e supra levoversão (nem todos os pacientes foram fotografados nas 4 posições citadas). Conferiu-se aos pacientes uma pontuação para a função do músculo oblíquo superior e músculo oblíquo inferior, que variou de -4 (negativo para hipofunção) a +4 (positivo para hiperfunção), ou 0 (normofuncionantes), antes e depois da edição das fotografias. Esses valores foram comparados à avaliação prévia atribuída pelos assistentes do estrabismo. Computou-se a diferença da pontuação entre eles em números naturais (inteiros e não negativos); foram calculadas média e desvio padrão dessas medidas.

**Resultado:**

A medida da maioria das fotos editadas apresentou média inferior as não editadas, à exceção de um paciente com hiperfunção de oblíquo superior esquerdo. Pacientes sem disfunção de oblíquos demonstraram, após edição das fotos, maior similaridade com o valor inicialmente determinado (p<0,05), assim como os pacientes com oblíquo superior direito hiperfuncionantes (p<0,01). Os mesmos resultados são encontrados nos pacientes com hipofunção dos oblíquos e hiperfunção de oblíquo inferior direito (p<0,01).

**Conclusão:**

O método utilizado para medida das funções musculares nos estrabismos verticais é reprodutível, acessível, simples, confiável, e confere maior uniformidade à aferição.

## INTRODUCTION

During binocular movement examination, the non-fixating eye is also observed. Binocular movements are classified as version or conjugated movement (both eyes targeting the same direction and side), and vergence or disjunctive movement (same direction but opposite sides). The versions include supraversion, infraversion, levoversion, and dextroversion, in addition to oblique movements such as supra-dextroversion, supra-levoversion, infra-dextroversion, and infra-levoversion. The vergence can be horizontal (convergence or divergence), vertical (positive or negative), or torsional (intorsion or extorsion)^(1,2)^.

In strabismus, a horizontal deviation that changes in an upor down-gaze is referred to as an “A” or “V” pattern (usually due to oblique muscle dysfunction). The “A” pattern is characterized by an accentuated convergence in up-gaze, while the “V” pattern shows marked convergence in down-gaze^(3-6)^. Some of the variants of these patterns, which are mostly uncommon and related to exotropia, are known such as lambda, diamond, and X^(6)^.

In the primary eye position (PEP), the visual axis is located outside the field of action of both oblique muscles, characterizing a complex action. The inferior oblique (IO) and superior oblique (SO) muscles have axes that are correspondingly 51° and 54° nasal to the Y-axis (coincidental with the visual axis in the PEP), respectively^(2)^. The SO and IO muscles have three actions in the PEP; the primary action is always more intense, and the others are considered secondary. In the PEP, the primary action of the SO muscle is intorsion, and the secondary actions are abduction and depression; the primary action of the IO muscle is extorsion, and its secondary actions are abduction and elevation^(1)^.

During examination of the oblique muscles, the patient targets an object in supraand infra-abduction, and the non-fixating eye is observed. In this position of supra-adduction and infra-adduction of the non-fixating eye, the oblique muscles perform a vertical motion. Thus, the vertical position of the fixating eye can be compared with the non-fixating eye in the supraand infra-adduction evaluations for hypofunction, normal function, or hyperfunction of the oblique muscles.

The versions must be interpreted with caution in patients who have undergone previous oblique muscle surgery and those with Graves’ disease, long-standing vertical strabismus, or dissociated vertical divergence (DVD), which can be associated with secondary superior rectus muscle contraction and restrictive strabismus. In these cases, the relative position of each eye may induce error in recognizing the muscles with hyperfunction or hypofunction.

Each evaluated muscle is given a score ranging from -4 to 4, with negative values indicating hypofunction; positive values, hyperfunction; and zero, near-to-normal function. However, this score is subjective and causes a different impression to each examiner. Therefore, the reproducibility of the technique is considered low. Strabismus measurement is paramount to defining clinical and surgical management, especially treatment planning.

To refine the version study and, consequently, the surgical planning and outcome, we developed a simple, accessible, and less subjective method for estimating deviations through the interposition of a grad system found in the photo editing program of any iPhone.

## METHODS

In this study, we propose to measure the reliability of a new test intended to evaluate and quantify oblique muscle function. This project was approved by the ethics and research committee of *Irmandade da Santa Casa de Misericórdia de São Paulo* (CAAE No. 81911317.0.0000.5479).

The smartphone iPhone by Apple has an IOS operational system that is frequently updated. Starting with the 9.0 version IOS, an app called “Photos” that allows brightness, contrast, exposition, and rotation adjustment of photographs was introduced.

In this study, 75 examiners (ophthalmologists, including current students and graduates) evaluated 22 photographs, registered in infraand supra-dextroversion, and infraand supra-levoversion (but not all patients were photographed in all 4 positions) from 9 selected patients from the Strabismus Outpatient Clinic of Santa Casa de São Paulo to analyze the oblique muscles. The inclusion criteria were patients of any age, sex, and ethnic ity, with or without oblique dysfunction, who could cooperate with the photograph registry. The exclusion criteria were patients who did not permit or collaborate with the examination and those who had undergone a previous oblique muscle surgery, Graves’ disease, long-standing vertical strabismus, or DVD.

These patients were examined by four staff members of the Strabismus Outpatient Clinic of Ophthalmology Department of Santa Casa de São Paulo, who jointly classified the oblique muscle function. A scale was used, ranging from -4 (hypofunction) to 4 (hyperfunction) or 0 (normal function). During submission of the photos to the 75 examiners, the criterion was already consolidated.

**Figure 1:**
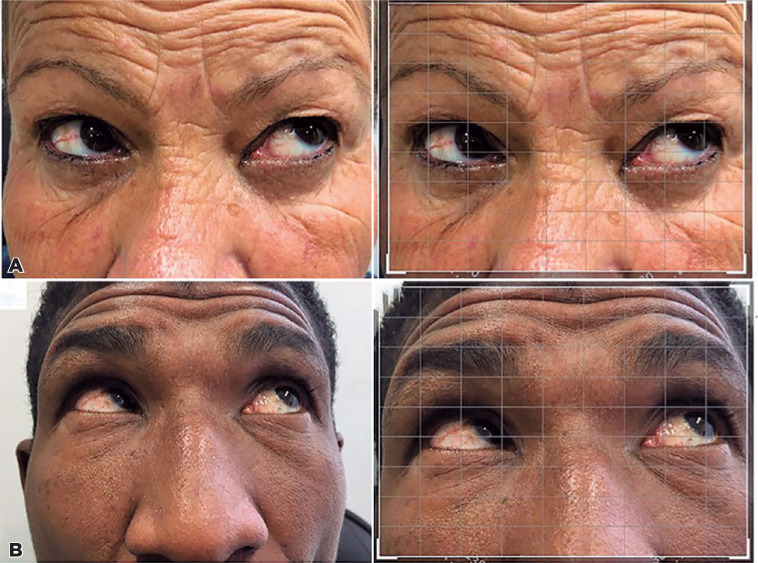
Patients with hypofunction in the right inferior oblique muscle (A, before editing and B, after editing).

Initially, one patient was photographed without right IO dysfunction; one, without left SO dysfunction; three, with right SO hyperfunction; two, with left SO hyperfunction; one, with left IO hypofunction; two, with right IO hypofunction (Figure 1); and one, with right IO hyperfunction. The examiners then classified the oblique muscle function by using the presented iPhone images, rating the function of these muscles from 1 to 4.

**Figure 2:**
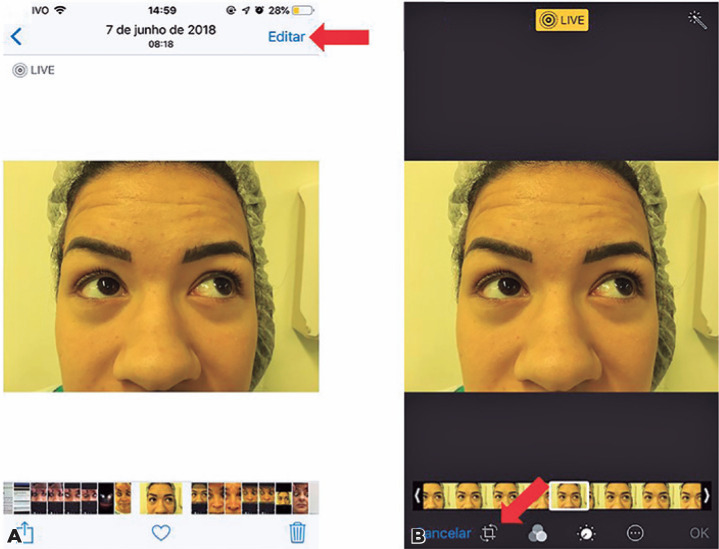
Screenshot showing how to edit the image with the iPhone Photos app. (A) The word “edit” was selected; (B) at the bottom left-hand corner, next to “cancel,” the edit icon was selected.

The photographs were edited by the researcher using the Photos iPhone app. After the photo was selected, in the top right-hand corner of the screen, the following events occurred:

Item 1: The word “edit” was selected; in the next screen, at the bottom left-hand corner, next to “cancel,” the edit icon was selected (Figure 2).

**Figure 3:**
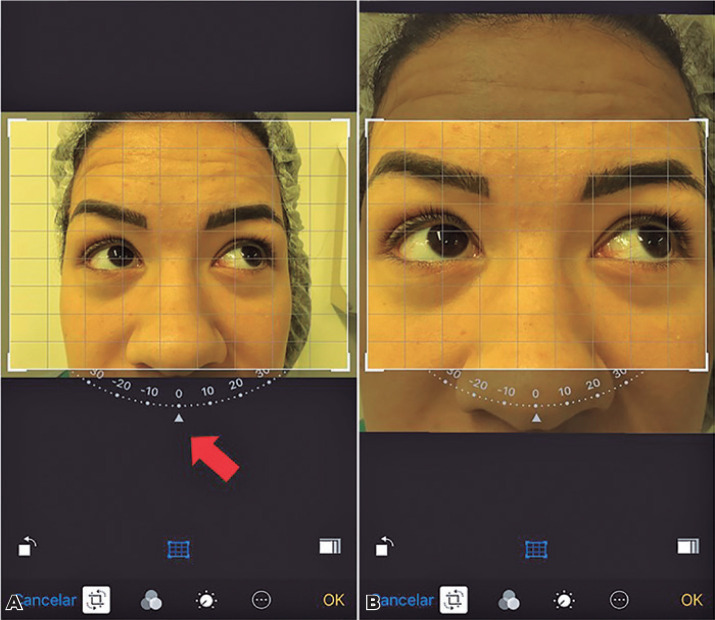
Screenshot showing how to edit the image on the iPhone Photos app. The photo shows formatting with a compass in the inferior margin. (A) As the compass is activated by pressing it, a grid image overlaps the photo. (B) The photo is rotated to align the horizontal lines with the limbus of the targeted eye.

Item 2: The photo showed formatting with a compass in the inferior margin. As the compass is activated by pressing it, a grid image overlapped the photo; the photo was rotated when necessary if the head was tilted. Next, the grade line was positioned always on the inferior limbus of the abducted eye/fixing eye in supra-levoversion and supra-dextroversion and on the superior limbus in the infra-levoversion and infra-dextroversion (Figure 3).

Item 3: With the finger over the compass, the screen was printed and saved in the album. All the photographs were erased when the research ended.

The edited photos were analyzed by the examiners, who again graded the evaluated oblique muscle function.

The classifications of the versions from the edited and non-edited photos were compared with the scores determined initially by the strabismus and orthoptic staff. The difference between the evaluations of the staff and examiners was registered as an absolute value (whole and non-negative). For instance, if the score given by the staff was -2 and that by an examiner was +1, the amount registered was 3.

Average and pattern deviations were calculated from the examiners’ measurements of the 22 evaluated images. A statistical analysis was performed using the Wilcoxon test.

## RESULTS

All of the photographs, edited and non-edited, were evaluated by 75 examiners. All the examiners invited to join the study could perform the proposed evaluation.

By using the Wilcoxon test, in patients without oblique disfunction, the first picture evaluated before and after editing presented a variation of *z*=-2,47 (p=0.01). The second picture showed a *z* score of -3.24 (p<0.01).

Variations of *z*=-3.51 (p<0.01), *z*=-3.90 (p<0.01), and *z*=-3.19 (p<0.01) were found in the first, second, and third pictures of the patients with right SO hyperfunction, respectively. In the patients with left SO hyperfunction, we did not find significant statistical variances. In the patients with left IO hypofunction, the variation was *z*=-2.83 (p<0,01). The patients with right IO hypofunction (attachment 1) presented with a *z* score of -4.00 (p<0.01) in the first picture and -6.97 (p<0.01) in the second picture. The patients with right IO hyperfunction displayed a variance of *z*=-3.32 (p<0.01).

## DISCUSSION

The study of ocular motility is crucial to the appropriate management of patients with strabismus. In cases with an “A” or “V” pattern, a preoperative version study is mandatory. The patient must target the fixating eye to an extreme version, which can be difficult if the patient fails to cooperate. Young children usually could not gaze or sustain an extreme gaze position for a long time for photography; moreover, delay between the moment of extreme gaze and the moment of photography, which can change the position of the eyes, is common and may induce error in the interpretation of oblique muscle dysfunction on the photograph. This causes a variability in the test. Therefore, in some patients, more than one photographic record was necessary until a photograph was taken with the best diagnostic position. However, these difficulties do not invalidate the usefulness of the tool.

No gold standard has been established for version examination; thus, the examination is subjective and, therefore, can mislead the analysis of hypofunction or hyperfunction (for instance, in cases presenting with ptosis). Still, the tool persists as the main technique for evaluating versions. In 2014, a study presented a method for quantifying ocular motility in 9 cardinal positions that requires no editing using Photoshop, with the limbus as reference^(7)^.

We developed a technique, which could be more accessible to specialists in strabology, to ease the evaluation of ocular motility for the assessment of oblique muscle dysfunction in vertical misalignments. The Photos app allows users to adjust the line (through compass rotation), taking the limbus of the contralateral eye as reference. This enables the adjustment of a patient’s head or smartphone rotation during the photograph.

The development of smartphones has a huge impact in ophthalmologic practice owing to its ever-increasing number of available apps, widespread accessibility, and low cost. In 2013, 342 ophthalmologic apps were available in the Apple App Store and Android Play Store for both physicians and patients^(8)^. In the field of strabology, apps that aid surgical planning, such as Strabismus Mobile (available in smartphones, limited to horizontal misalignments) and the software SquintMaster (Singh, 2008), were developed to suggest a diagnosis and propose a surgical plan^(9)^; however, these depend on the previous deviation admeasurement, which is obtained subjectively. The use of smartphones to obtain general ophthalmologic photographs has become popular^(8)^. EyeTilt is another app available in the Apple App Store, which can be used in strabismology to measure head tilt objectively during patient examination or with photos taken from patients. In a recent study, the Photos app of iPhone was also used to measure head tilt using photographs from patients, with good results^(10)^.

Considering the results of this study, measurement using only one of the edited photographs (patient with left SO hyperfunction) demonstrated an average value superior to that from the non-edited photo, while the other photos showed inferior average values (Table 1).

**Table 1:** Evaluation of images before and after editing

	Mean	Pattern deviation	Minimum	Maximum	z	p Value
RIO NF BE	0.53	684	0	2		
RIO NF AE	35	507	0	2	-2,475^a^	0.013
LSO NF BE	0.93	0.759	0	3		
LSO NF AE	0.63	0.564	0	2	-3,245^a^	0.001
RSO HEF +1 BE	0.71	0.653	0	3		
RSO HEF +1 AE	0.39	0.543	0	2	-3,507^a^	0.000
RSO HEF + 2 BE	1.45	1.031	0	4		
RSO HEF + 2 AE	0.83	0.921	0	3	-3,900^a^	0.000
RSO HEF +1 BE	1.69	0.788	0	3		
RSO HEF +1 AE	1.32	0.796	0	3	-3,192^a^	0,001
LSO HEF +2 BE	0.72	0.781	0	4		
LSO HEF +2 AE	0.68	0.681	0	3	-0.209^a^	0.835
LSO HEF +2 BE	0.84	0.772	0	4		
LSO HEF +2 AE	0.89	0.751	0	3	-0.985^b^	0.325
LIO HOF -2 BE	0.79	0.722	0	3		
LIO HOF -2 AE	0.49	0.623	0	2	-2,826^a^	0.005
RIO HOF -2 BE	0.81	0.896	0	4		
RIO HOF -2 AE	0.37	0.540	0	2	-3,996^a^	0.000
RIO HOF -1 BE	1.13	0.844	0	5		
RIO HOF -1 AE	0.09	0.411	0	3	-6,966^a^	0.000
RIO HEF +2 BE	0.87	0.684	0	3		
RIO HEF +2 AE	0.49	0.645	0	2	-3,325^a^	0.001

*RIO*= right inferior oblique; *LIO*= left inferior oblique; *LSO*= left superior oblique; *RSO*= right superior oblique; *NF=* normal function; *HEF*= hyperfunction; *HOF*= hypofunction; *BE*= before editing; *AE*= after editing; *z*= *z* score obtained using a Wilcoxon test.

a = Based on positive ranks.

b = Based on negative ranks.

The scores of the patients without oblique dysfunction as evidenced after photo editing showed greater similarity with the initially determined scores (p<0,05), like those in patients with right SO hyperfunction (p<0.01). The same results were found in the patients with oblique hypofunction and right IO hyperfunction (p<0.01; Table 1).

This study has some limitations such as the subjective method with which the previously established score was determined. This may be the reason why the patient with left SO hyperfunction showed no change in behavior, as the patient already had hyperfunction before the present assessment. However, the interposition of grids eases the squint perception and minimizes the discrepancy between the examiners.

This study can be expanded by introducing greater variabilities in hypofunction and hyperfunction and increasing the number of examiners.

In conclusion, the presented method for version admeasurement in vertical strabismus is reproducible, accessible, simple, and reliable, and improves the consistency of ocular motility measurement.
